# The associations between the *MAPT* polymorphisms and Alzheimer’s disease risk: a meta-analysis

**DOI:** 10.18632/oncotarget.16490

**Published:** 2017-03-22

**Authors:** Futao Zhou, Danli Wang

**Affiliations:** ^1^ College of Medicine & Health, Lishui University, Lishui Zhejiang, China

**Keywords:** Alzheimer’s disease, microtubule-associated protein tau, single nucleotide polymorphisms, meta-analysis

## Abstract

Published studies revealed that the microtubule-associated protein *tau* (*MAPT*) gene polymorphisms increased Alzheimer’s disease (AD) risk; the associations of 4 single nucleotide polymorphisms (SNPs, rs242557G/A, rs2471738C/T, rs3785883G/A and rs1467967A/G) of the *MAPT* gene with AD risk, however, remain inconclusive. Here, we conducted a meta-analysis to investigate the relationship between the *MAPT* SNPs and AD risk. A significant association of SNP rs242557 with AD risk was found in a dominant [odds ratio (OR) = 1.05, 95% confidence interval (CI) = 1.01, 1.10, *P* = 0.025] genetic model, and a suggestive association in an allelic (OR = 1.03, 95% CI = 1.00, 1.06, *P =* 0.078). When *APOE* epsilon 4 carrier status was included in stratified analysis, this association was even stronger (allelic model for the *APOE* epsilon 4 positive individuals: OR = 1.24, 95% CI = 1.08, 1.43, *P* = 0.003). Furthermore, a significant association of SNP rs2471738 with AD risk was found under all the four models (allelic: OR = 1.11, 95% CI = 1.01, 1.20, *P =* 0.021; dominant: OR = 1.10, 95% CI = 1.00, 1.21, *P* = 0.046; recessive: OR = 1.18, 95% CI = 1.05, 1.32, *P =* 0.004; additive: OR = 1.20, 95% CI = 1.07, 1.34, *P =* 0.002) models. However, pooled results suggest that the neither rs3785883 nor rs1467967 is associated with AD risk under all the four genetic models. In summary, our study provides further evidence of the associations of the *MAPT* SNPs with AD risk.

## INTRODUCTION

One of the neuropathological hallmarks of Alzheimer's Disease (AD) is the neurofibrillary tangle, which contains paired helical filaments (PHFs) composed of hyperphosphorylated forms of the microtubule-associated protein tau (MAPT) by mechanism which is not illustrated [1]. Increasing attention has been paid to endogenous and exogenous factors, as well as genetic risk factors contributing to the incidence of AD [2], stimulating the disease progression of AD [3]. It was believed that the identification of key genetic determinants for AD might help further understand its underlying mechanism.

Human *MAPT* gene is located on chromosome 17q21. There have been conflicting results showing positive or negative findings on the association between the *MAPT* SNPs and AD risk. Some studies were showed that SNPs rs242557 [4, 5], rs3785883 [6] in US series, rs2471738 [6, 7] and rs1467967 [8] of the *MAPT* gene might been associated with increased AD risk. Some studies were, however, reported that rs242557 [8–10], rs3785883 [11–14], rs2471738 [11, 14, 15] and rs1467967 [7, 16] might not be associated with AD risk [10, 11, 13, 16, 17].

There are many factors leading to these different results about the association between the *MAPT* SNPs and AD risk. One of primary reasons is low statistical power and the limited sample size in each study. Therefore, we performed a meta-analysis on the association between the *MAPT* SNPs and AD risk by pooling all available published data. In this study, we evaluated the genetic heterogeneity of the studies included and then carried out a meta-analysis on the association between the *MAPT* SNPs (rs242557, rs2471738, rs3785883 and rs1467967) with AD risk to make a more accurate assessment of the relationship for greater power in detecting the disease associations.

## RESULTS

### Characteristic of eligible studies

**Figure 1:**
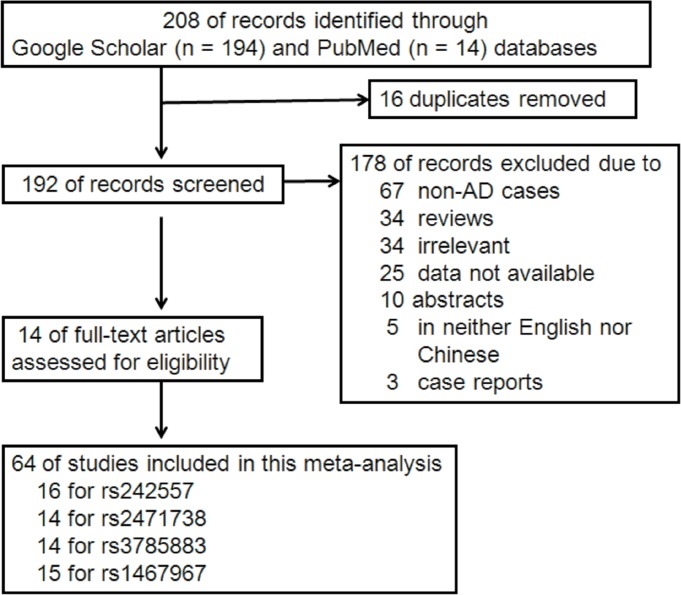
Flow diagram of study selection

The literature search was done on studies up to January 2017 and availability of an English-language abstract or paper for review; this yielded 208 hits (PubMed: 14, Google scholar: 194). 194 of these were excluded, including 16 duplicates, 67 non-AD case reports, 34 reviews, 34 irrelevant studies, 25 data not available, 10 abstracts, 5 non-English language papers (also non-Chinese) and 3 case reports. In total, 64 independent studies from 14 articles published from 2005 to 2016 providing data of the *MAPT* genotype, were included in the current meta-analysis (16 for rs242557, 14 for rs2471738, 14 for rs3785883 and 15 for rs1467967; Figure 1). We found that in all the studies included SNPs neither rs75721 (within exon 14) nor rs9468 (within exon 13) was significantly associated with increased AD risk (results not shown). So, we analyzed the associations between these SNPs (rs242557, rs2471738, rs3785883 and rs1467967) of the *MAPT* gene and AD risk involved in 14666/17532, 13812/17201, 14607/17883 and 15064/17687 cases/controls, respectively. The NOS results indicated that the methodological quality of these selected studies was generally good. The study characteristics were listed in Table 1.

**Table 1:** Main characteristics of the studies included in this meta-analysis of the associations between these SNPs of the *MAPT* gene and AD risk

SNP loci	Allele	Ref no.	First author	Year	Country	Case (n)	CTR (n)	Case genotype			Control genotype			HWE_ct_	NOS
								AA	AB	BB	AA	AB	BB		
rs242557 Exon 1	G > A	11	Abraham, R.	2009	UK	979	1139	144	456	379	143	563	433	0.054	8
		13	Allen, M.(Mayo Cohort)	2014	USA	1802	3133	260	838	704	500	1373	1260	**0.0001**	8
		13	Allen, M.(ADGC Cohort)	2014	USA	6705	6702	865	3082	2758	849	3081	2772	0.88	8
		13	Allen, M.(JS)	2014	USA	828	932	124	386	318	141	404	387	**0.04**	8
		13	Allen, M.(RS)	2014	USA	460	2201	60	215	185	359	969	873	**0.001**	8
		8	Feulner, T. M.	2010	Germany	491	479	68	246	177	81	220	178	0.36	9
		9	Huin, V.	2016	France	35	19	5	16	14	3	9	7	0.97	7
		5	Laws, S. M.	2007	Germany	434	279	64	205	165	28	120	131	0.99	8
		4	Liu, Q. Y.	2013	China	796	796	146	394	256	134	356	306	0.08	8
		10	Mateo, I.(a)	2008	Spain	300	360	30	127	143	36	153	171	0.84	8
		16	Mukherjee, O.	2007	USA	361	358	47	166	148	49	167	142	0.99	6
		6	Myers, A. J.(US)	2005	USA	181	131	26	85	70	12	55	64	0.97	8
		6	Myers, A. J.(UK)	2005	UK	179	121	32	87	60	15	55	51	0.98	8
		6	Myers, A. J. (US)	2007	USA	296	128	36	135	125	17	60	51	0.92	8
		6	Myers, A. J. (US/UK)	2007	UK	655	380	94	309	252	44	171	165	0.98	8
		12	Seto-Salvia, N.	2011	Spain	164	374	19	74	71	38	163	173	0.97	8
	Total					14666	17532								
	APOE(+)	4	Liu, Q. Y.	2013	China	200	122	44	96	60	18	52	52	0.403	8
		6	Myers, A. J. (US/UK)	2005	USA	360	252	55	171	134	34	105	113	0.229	8
		11	Abraham, R.	2009	UK	597	271	83	271	243	21	139	111	**0.012**	8
	APOE(−)	4	Liu, Q. Y.	2013	China	596	674	102	298	196	116	304	254	0.1291	8
		6	Myers, A. J. (US/UK)	2005	USA	360	252	64	175	121	26	109	117	0.9342	8
	Total					2113	1571								
rs2471738 Intron 9	C > T	11	Abraham, R.	2009	UK	970	1125	50	333	587	61	388	676	0.59	8
		13	Allen, M.(Mayo Cohort)	2014	USA	1980	3302	106	671	1203	135	1112	2055	0.31	8
		13	Allen, M.(ADGC Cohort)	2014	USA	6942	7239	292	2265	4385	287	2315	4637	0.93	8
		13	Allen, M.(JS)	2014	USA	851	947	39	297	515	28	334	585	**0.02**	8
		13	Allen, M.(RS)	2014	USA	585	2355	33	194	358	107	778	1470	0.75	8
		14	Chang, C. W.	2014	China	109	108	6	38	65	7	40	61	0.9	7
		16	Mukherjee, O.	2007	USA	361	358	13	111	237	16	119	223	0.98	6
		15	Mateo, I.(b)	2008	Spain	293	396	8	84	201	9	110	277	0.62	8
		6	Myers, A. J.(US)	2005	USA	181	131	12	70	99	4	39	88	0.9	8
		6	Myers, A. J.(UK)	2005	UK	179	121	10	65	104	4	36	81	1	8
		6	Myers, A. J. (US)	2007	USA	296	128	14	102	180	2	31	95	0.77	8
		6	Myers, A. J. (US/UK)	2007	UK	655	380	38	239	378	10	102	268	0.94	8
		12	Seto-Salvia, N.	2011	Spain	164	374	3	41	120	12	109	253	0.95	8
		19	Vazquez-Higuera, J. L.	2009	Spain	246	237	9	64	173	5	62	170	0.81	8
Total						13812	17201								
rs3785883 Intron 3	G > A	11	Abraham, R.	2009	UK	967	1139	29	272	666	33	332	774	0.72	8
		13	Allen, M.(Mayo Cohort)	2014	USA	1954	3293	66	581	1307	110	982	2201	0.97	8
		13	Allen, M.(ADGC Cohort)	2014	USA	7397	7790	254	2135	5008	235	2203	5352	0.65	8
		13	Allen, M.(JS)	2014	USA	841	943	30	238	573	26	267	650	0.82	8
		13	Allen, M.(RS)	2014	USA	578	2350	23	176	379	84	715	1551	0.89	8
		14	Chang, C. W.	2014	China	108	108	3	31	74	2	26	80	0.95	7
		8	Feulner, T. M.	2010	Germany	491	479	28	148	315	21	133	325	0.12	9
		5	Laws, S. M.	2007	Germany	433	279	11	118	304	11	88	180	0.97	8
		16	Mukherjee, O.	2007	USA	361	358	14	116	231	14	115	229	0.93	6
		6	Myers, A. J.(US)	2005	USA	181	131	5	51	125	6	45	80	0.92	8
		6	Myers, A. J.(UK)	2005	UK	181	131	3	41	137	3	33	95	0.95	8
		6	Myers, A. J. (US)	2007	USA	296	128	12	95	189	2	27	99	0.92	8
		6	Myers, A. J.(US/UK)	2007	UK	655	380	19	185	451	11	107	262	0.98	8
		12	Seto-Salvia, N.	2011	Spain	164	374	6	51	107	17	124	233	0.92	8
Total						14607	17883								
rs1467967 Exon 1	A > G	11	Abraham, R.	2009	UK	982	1153	88	417	477	93	509	551	0.1	8
		13	Allen, M.(Mayo Cohort)	2014	USA	1868	3118	220	812	836	340	1376	1402	0.93	8
		13	Allen, M.(ADGC Cohort)	2014	USA	7110	7255	765	3151	3194	752	3232	3271	0.26	8
		13	Allen, M.(JS)	2014	USA	831	905	91	372	368	85	408	412	0.27	8
		13	Allen, M.(RS)	2014	USA	536	2213	70	241	225	255	968	990	0.43	8
		14	Chang, C. W.	2014	China	108	108	17	52	39	14	50	44	0.97	7
		21	Elias-Sonnenschein, L. S.	2013	Finnish	869	685	104	391	374	89	308	288	0.64	8
		8	Feulner, T. M.	2010	Germany	491	479	56	228	207	47	191	241	0.31	9
		5	Laws, S. M.	2007	Germany	433	279	47	192	194	39	131	109	0.97	8
		6	Myers, A. J.(US)	2005	USA	181	131	18	79	84	19	62	50	0.98	8
		6	Myers, A. J.(UK)	2005	UK	179	121	18	78	83	13	54	54	0.93	8
		6	Myers, A. J. (US)	2007	USA	296	128	32	131	133	15	57	56	0.93	8
		6	Myers, A. J.(US/UK)	2007	UK	655	380	71	290	294	47	173	160	0.98	8
		16	Mukherjee, O.	2007	USA	361	358	42	162	157	46	165	147	0.98	6
		12	Seto-Salvia, N.	2011	Spain	164	374	14	67	83	26	145	203	0.99	8
Total						15064	17687								

Abbreviations: Ref no: reference number; NOS, the Newcastle-Ottawa Scale; CTR, control; HWEct, Hardy-Weinberg Equilibrium in controls;

### Heterogeneity test

The strength of the association was estimated in the allelic, dominant, recessive and additive models. The heterogeneity among studies was tested with Q statistic and further quantified by *I*^2^ statistic. As measured by the *I*^2^ (Table 2), in this meta-analysis no significant heterogeneity existed between studies under all the genetic models tested for rs242557 (the range of *I*^2^ values from 0 to 33.1%), rs3785883 (the range of *I*^2^ values from 0 to 29.1%), and rs1467967 (the range of *I*^2^ values from 0 to 17.5%). Therefore, the fixed-effect model (Mantel-Haenszel method) was used to calculate the pooled ORs. However, for rs2471738 there was significant heterogeneity observed between studies under the allelic and dominant models (*I*^2^ = 62.0 and 57.1 for the allelic and dominant genetic models, respectively). Therefore, the random-effect model (Inverse Variance method) was used to calculate the pooled ORs under allelic and dominant models (fixed-effect model for the recessive and additive genetic models).

**Table 2:** The genetic heterogeneity test

			Genetic model		*X*^2^	*p*	*I*^2^ (%)
rs242557	NO stratification		Allelic	A *vs*. G	22.42	0.097	33.1
			Dominant	AA+AG *vs*. GG	18.94	0.216	20.8
			Recessive	AA *vs*. AG+GG	18.50	0.237	18.9
			Additive	AA *vs*. GG	21.35	0.126	29.8
	Stratified by *APOE* ε4 allele	Positive	Allelic	A *vs*. G	1.87	0.393	0
		negative	Allelic	A *vs*. G	5.4	0.02	81.5
rs2471738			Allelic	T *vs*. C	34.21	0.001	62.0
			Dominant	TT+TC *vs*. CC	30.32	0.004	57.1
			Recessive	TT *vs*. TC+CC	14.68	0.328	11.5
			Additive	TT *vs*. CC	18.39	0.143	29.3
rs3785883			Allelic	A *vs*. G	18.33	0.146	29.1
			Dominant	AA+AG *vs*. GG	16.29	0.234	20.2
			Recessive	AA *vs*. AG+GG	6.17	0.94	0
			Additive	AA *vs*. GG	7.97	0.846	0
rs1467967			Allelic	G *vs*. A	16.96	0.258	17.5
			Dominant	GG+AG *vs*. AA	15.12	0.37	7.4
			Recessive	GG *vs*. AG+AA	8.7	0.85	0
			Additive	GG *vs*. AA	13.19	0.512	0

### Meta-analysis results of the association between SNP rs242557 and AD risk

**Figure 2:**
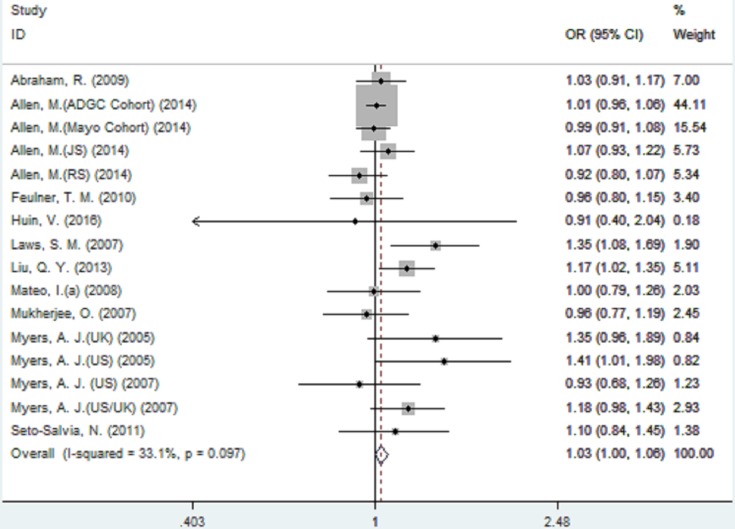
Forest plot for the meta-analysis of the association of SNP rs242557 and AD risk under the allelic model (A *vs*. **G**)

**Figure 3:**
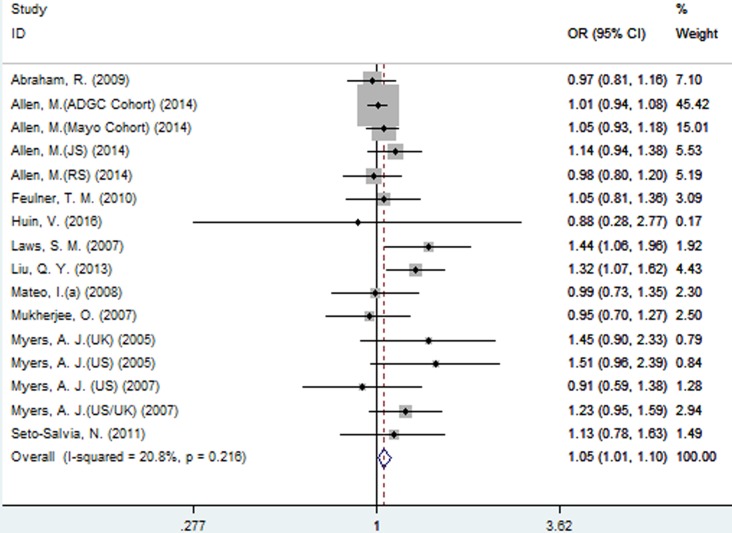
Forest plot for the meta-analysis of the association of SNP rs242557 and AD risk under the dominant model (AA + AG *vs*. **GG**)

For rs242557 when the 16 studies were pooled into the meta-analysis using the fixed-effect model, a significant association was observed under the dominant (OR = 1.05, 95% CI = 1.01, 1.10, *P* = 0.025, Figure 3) model, and there was a trend under the allelic (OR = 1.03, 95% CI = 1.00, 1.06, *P =* 0.078, Figure 2) model. However, no significant association was found under the recessive (OR = 1.06, 95% CI = 0.95, 1.08, *P* = 0.766) and additive models (OR = 1.04, 95% CI = 0.97, 1.12, *P* = 0.223).

**Figure 4:**
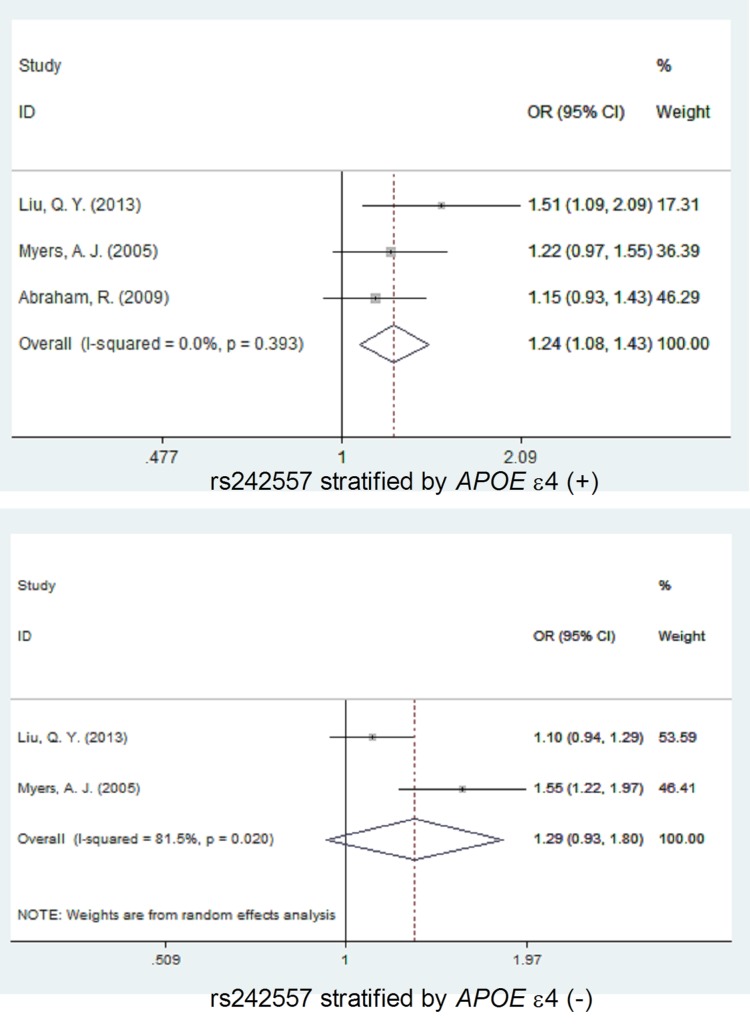
Forest plot for the meta-analysis of the association of SNP rs242557 and AD risk stratified by *APOE* ε4 allele status

When stratified by *APOE* ε4 carrier status, the association between the rs242557 SNP and AD risk was observed to be stronger in the individuals with *APOE* ε4-positive genotype (with no heterogeneity, *I*^2^ = 0, OR = 1.24, 95% CI = 1.08, 1.43, *P* = 0.003) than without stratification (OR = 1.03, 95% CI = 1.00, 1.06, *P =* 0.078) under the allelic model. But for the individuals with *APOE* ε4-negative genotype (*APOE* ε4-), there was large heterogeneity (*I*^2^ = 81.5, Table 2) under the allelic model, and no significant association between the rs242557 SNP with AD risk (OR = 1.29, 95% CI = 0.93, 1.80, *P* = 0.132, Table 3, Figure 4).

**Table 3:** The pooled results of the associations between these SNPs and AD risk as well as publication bias evaluation of the studies included

SNP locus		Genetic model		Effect model	*P*z	Pooled OR	95% CI	Publication bias (*p* value)	
								Begg’s	Egger’s
rs242557		Allelic	A vs. G	Fixed	**0.078**	**1.03**	**1.00-1.06**	0.753	0.982
		Dominant	AA+AG vs. GG	Fixed	**0.025**	**1.05**	**1.01-1.10**	0.753	0.933
		Recessive	AA vs. AG+GG	Fixed	0.766	1.06	0.95-1.08	0.558	0.341
		Additive	AA vs. GG	Fixed	0.223	1.04	0.97-1.12	0.558	0.337
	APOE (+)	Allelic	A vs. G	Fixed	**0.003**	**1.24**	**1.08-1.43**	0.296	0.371
	APOE (−)	Allelic	A vs. G	Random	0.132	1.29	0.93-1.80	1.0	-
rs2471738		Allelic	T vs. C	Random	**0.021**	**1.11**	**1.01-1.20**	0.827	0.493
		Dominant	TT+TC vs. CC	Random	**0.046**	**1.10**	**1.00-1.21**	0.101	0.667
		Recessive	TT vs. TC+CC	Fixed	**0.004**	**1.18**	**1.05-1.32**	0.869	0.589
		Additive	TT vs. CC	Fixed	**0.002**	**1.20**	**1.07-1.34**	0.189	0.469
rs3785883		Allelic	A vs. G	Fixed	0.179	1.03	0.99-1.07	0.324	0.543
		Dominant	AA+AG vs. GG	Fixed	0.32	1.02	0.98-1.07	0.189	0.067
		Recessive	AA vs. AG+GG	Fixed	0.144	1.10	0.97-1.24	0.274	0.732
		Additive	AA vs. GG	Fixed	0.126	1.10	0.97-1.25	0.101	0.051
rs1467967		Allelic	G vs. A	Fixed	0.447	1.01	0.98-1.05	0.767	0.830
		Dominant	GG+AG vs. AA	Fixed	0.737	1.01	0.96-1.05	0.921	0.804
		Recessive	GG vs. AG+AA	Fixed	0.276	1.04	0.97-1.12	0.553	0.572
		Additive	GG vs. AA	Fixed	0.301	1.04	0.97-1.12	0.692	0.383

### Meta-analysis results of the association between SNP rs2471738 and AD risk

**Figure 5:**
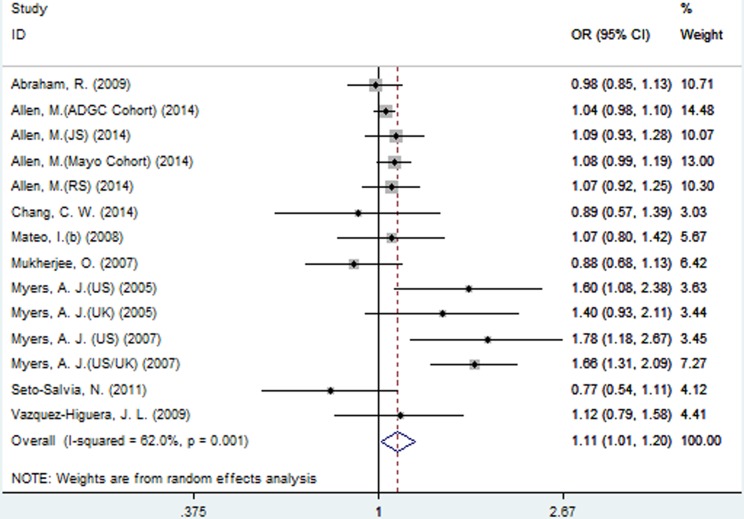
Forest plot for the meta-analysis of the association of SNP rs2471738 and AD risk under the allelic model (T *vs*. **C**)

**Figure 6:**
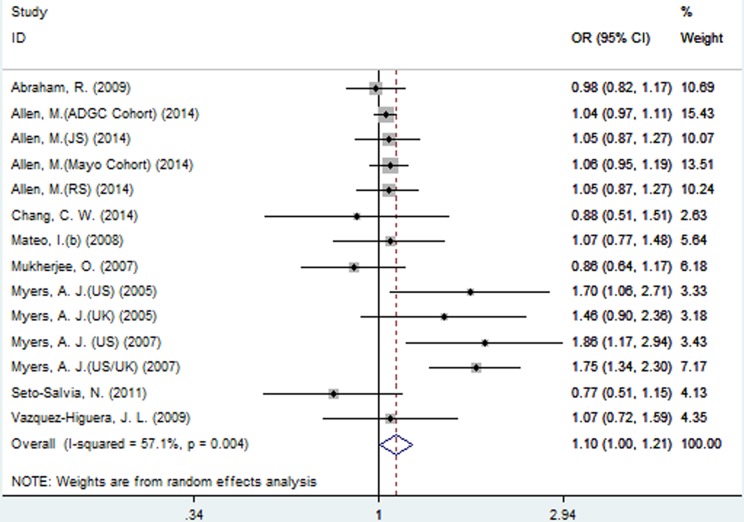
Forest plot for the meta-analysis of the association of SNP rs2471738 and AD risk under the dominant model (TT + TC *vs*. **CC**)

**Figure 7:**
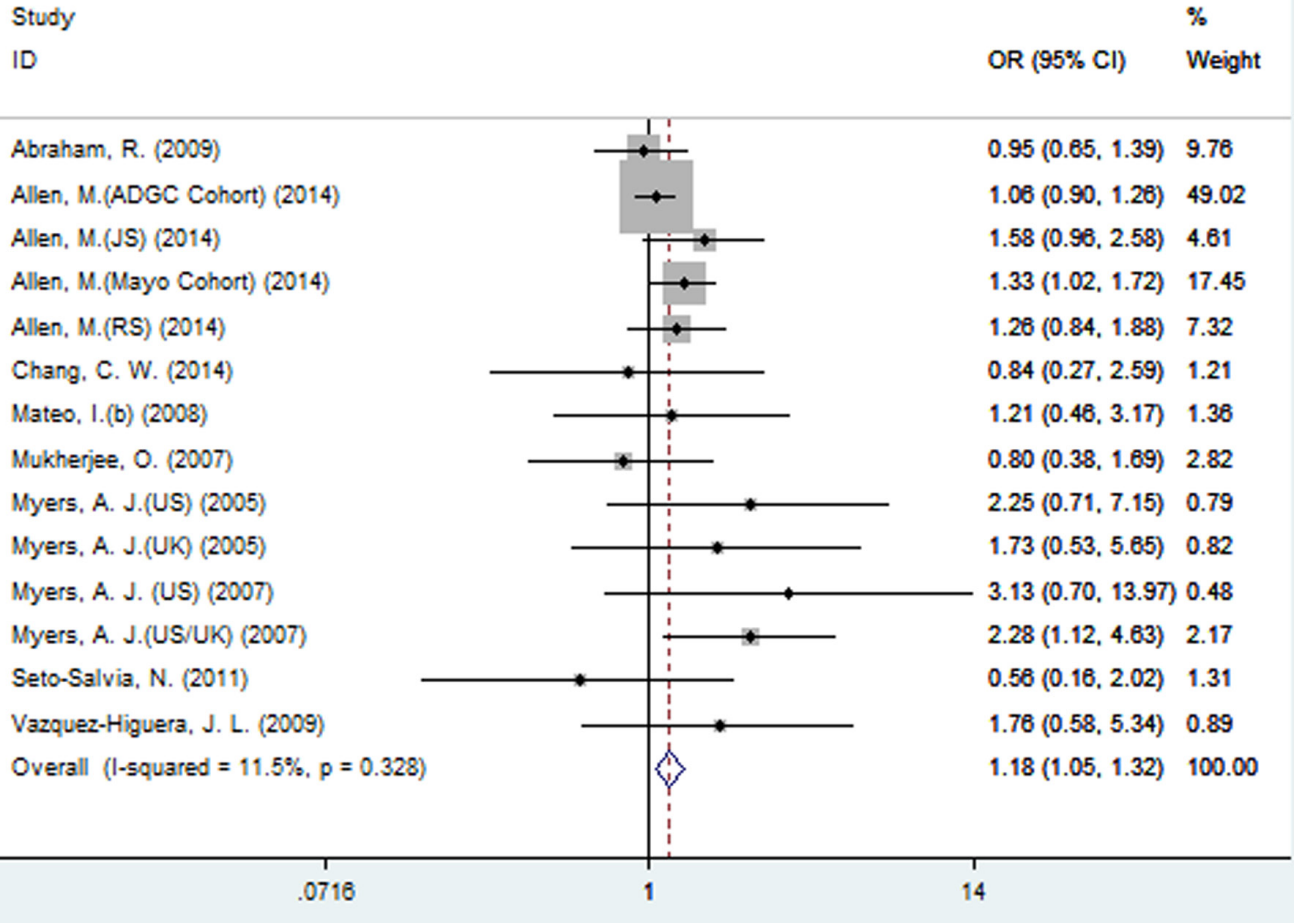
Forest plot for the meta-analysis of the association of SNP rs2471738 and AD risk under the recessive model (TT *vs*. **CC + TC**)

**Figure 8:**
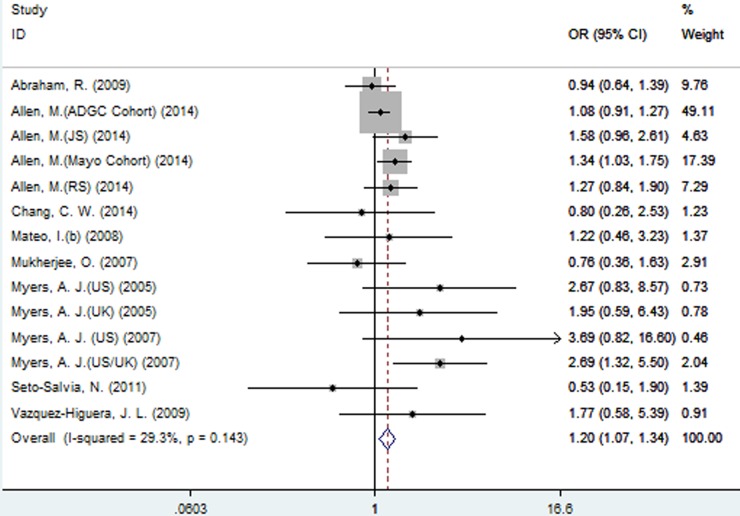
Forest plot for the meta-analysis of the association of SNP rs2471738 and AD risk under the additive model (TT *vs*. **CC**)

A significant association between SNP rs2471738 and AD risk was identified under the allelic (random-effect, OR = 1.11, 95% CI = 1.01, 1.20, *P* = 0.021, Figure 5 and Table 3) and dominant (OR = 1.10, 95% CI = 1.00, 1.21, *P* = 0.046, Figure 6 and Table 3) models. A significant association between SNP rs2471738 and AD risk was also identified under the recessive (fixed-effect, OR = 1.18, 95% CI = 1.05, 1.32, *P* = 0.004, Figure 7 and Table 3) and additive (OR = 1.20, 95% CI = 1.07, 1.34, *P* = 0.002, Figure 8 and Table 3) models.

### Meta-analysis results of the associations between SNPs rs3785883 and rs1467967 and AD risk

**Figure 9:**
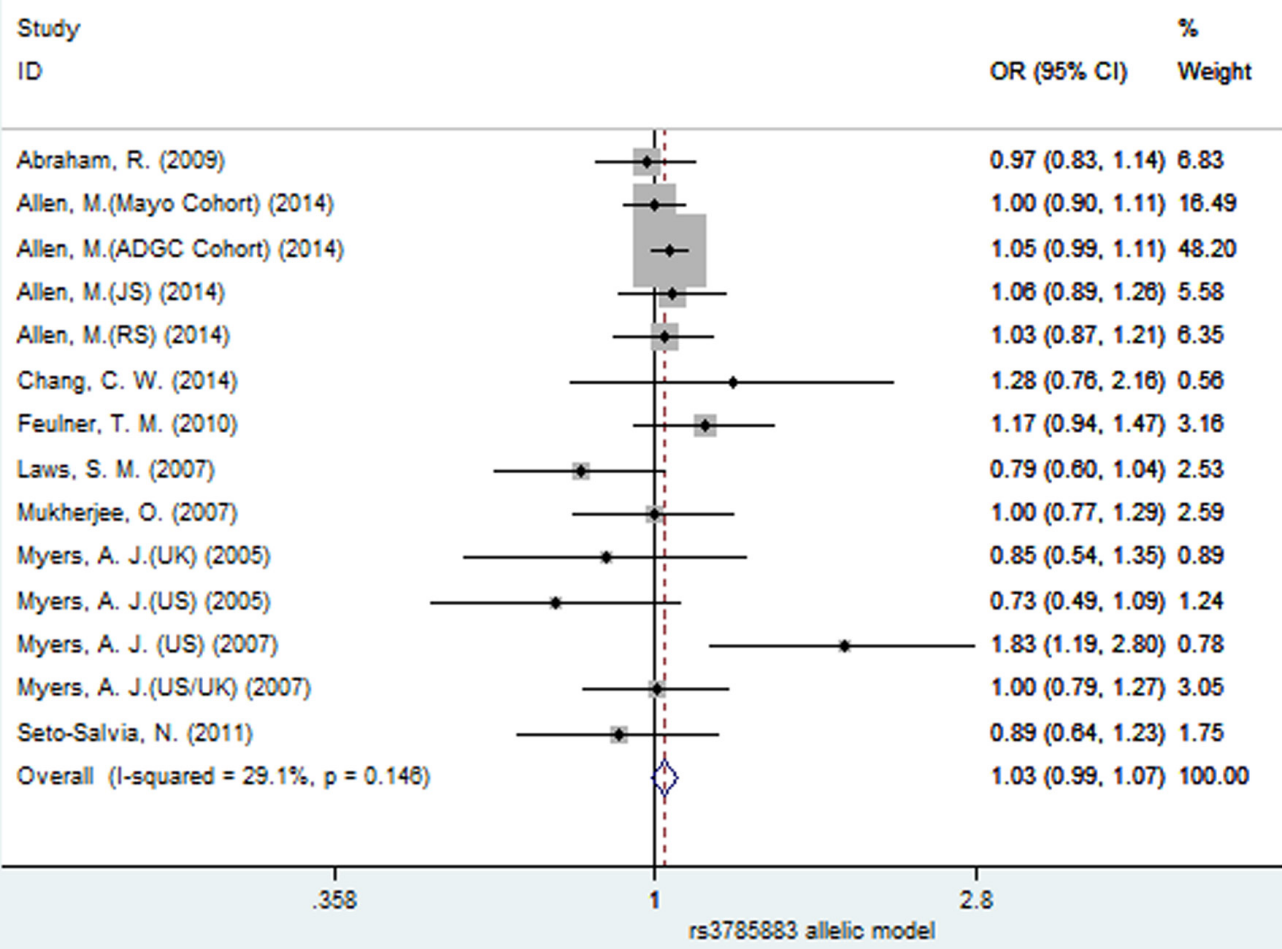
Forest plot for the meta-analysis of the association of SNP rs3785883 and AD risk under the allelic model (A *vs*. **G**)

Using fixed-effect model, no significant association between SNP rs3785883 and AD risk was observed under all the four models (allelic: OR = 1.03, 95% CI = 0.99, 1.07, *P =* 0.179, Figure 9; dominant: OR = 1.02, 95% CI = 0.98, 1.07, *P =* 0.32; recessive: OR = 1.10, 95% CI = 0.97, 1.24, *P =* 0.144; additive: OR = 1.10, 95% CI = 0.97, 1.25, *P =* 0.126, Table 3).

**Figure 10:**
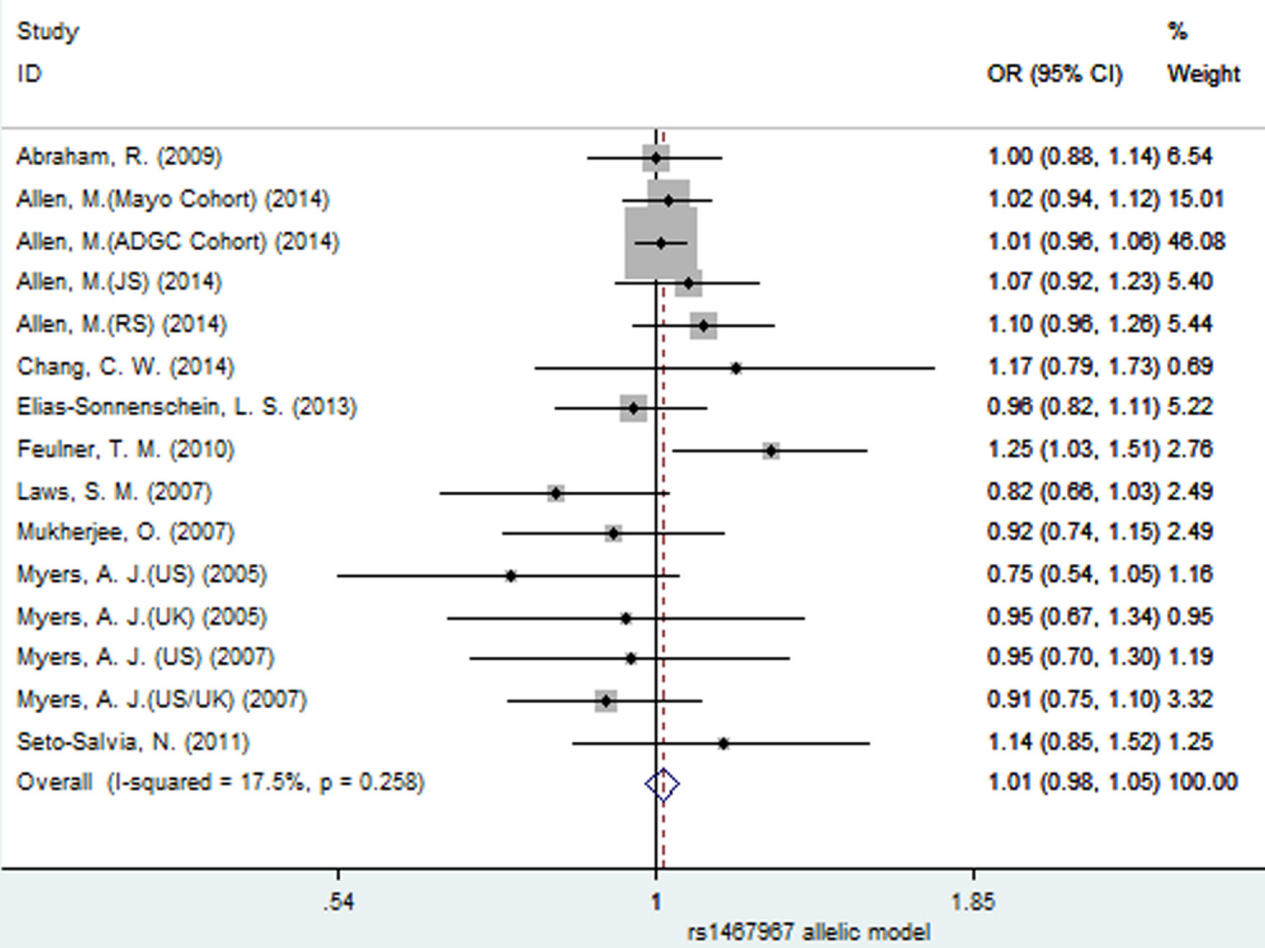
Forest plot for the meta-analysis of the association of SNP rs1467967 and AD risk under the allelic model (G *vs*. **A**)

Similarly, no significant association between SNP rs1467967 and AD risk was found under all the four models (fixed-effect, allelic: OR = 1.01, 95% CI = 0.98, 1.05, *P =* 0.449, Figure 10; dominant: OR = 1.01, 95% CI = 0.96, 1.05, *P =* 0.737; recessive: OR = 1.04, 95% CI = 0.97, 1.12, *P =* 0.276; additive: OR = 1.04, 95% CI = 0.97, 1.12, *P =* 0.301, Table 3).

### Sensitivity analysis and evaluation of publication bias

**Figure 11:**
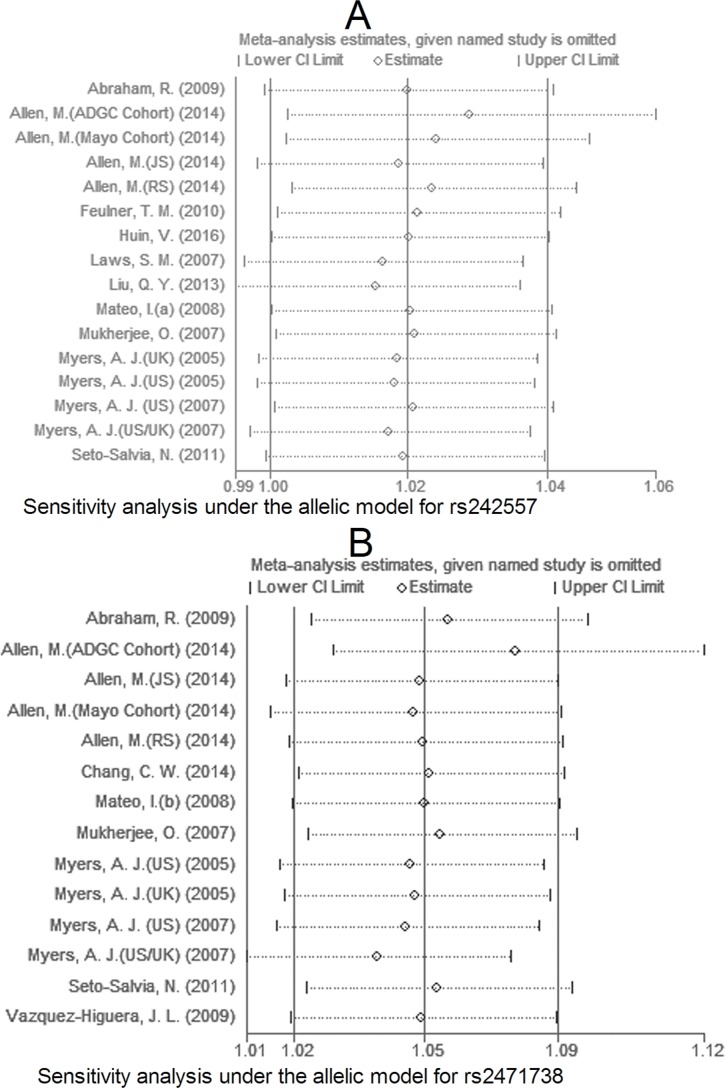
Sensitivity analysis for rs242557 A. and rs2471738 B. under the allelic model

**Figure 12:**
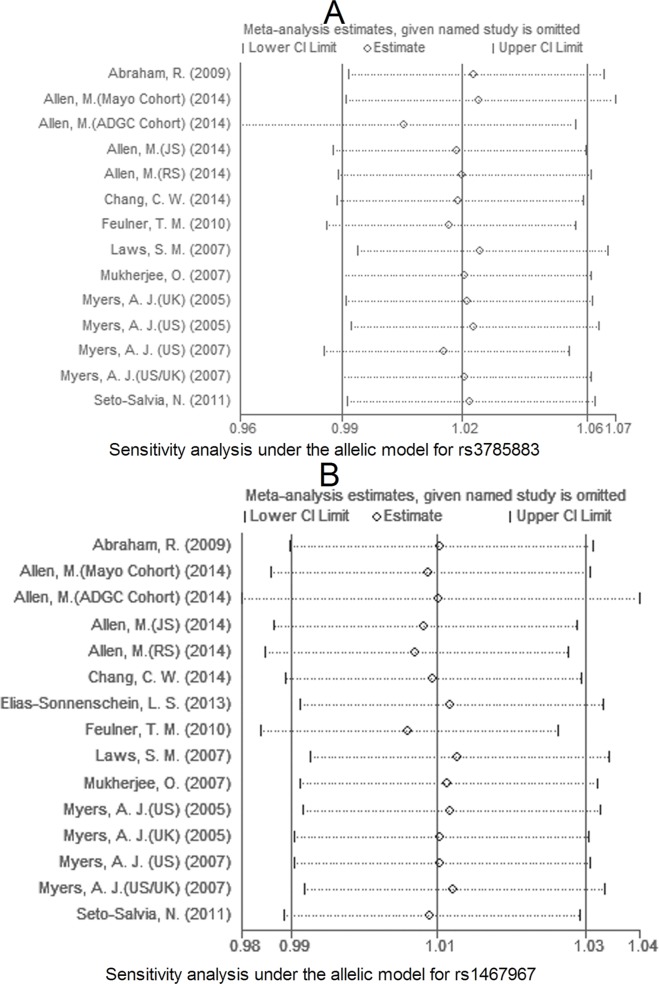
Sensitivity analysis for rs3785883 A. and rs1467967 B. under the allelic model

Due to large heterogeneity between studies for rs2471738, we performed a sensitivity analysis by excluding a study [Allen, M. (JS), 2014; see Table 1] with departure from Hardy-Weinberg Equilibrium (HWE) in controls, we did not observe increased homogeneity across the rest studies (data not shown), suggesting that HWE deviation was not a source of between-study heterogeneity. The sensitivity analysis showed that for rs242557 and rs2471738 none of the studies included significantly changed the results under the allelic model (Figure 11A and 11B, respectively). The same results were observed for rs3785883/rs1467967 (Figure 12 A and 12B, respectively). Begg's and Egger's test were used to estimate the severity of publication bias with a *P-value* < 0.05 being considered statistically significant. No evidence of publication bias was found in any genetic model (Table 3).

## DISCUSSION

Tau protein is specifically expressed in neurons, directly interacts with tubulin and mediates its assembly [18]. It was found that the *MAPT* rs242557 (within exon 1) SNP was significantly associated with late-onset AD in 1592 Han Chinese subjects [4], in the German population [5] and in the US series [7]. However, it was reported that this SNP was not significantly associated with AD risk [10, 11] in the UK series [7]. For the rs2471738 (within intron 9) SNP, study findings revealed that there was significant association in the US series [6, 7] and US/UK series [6], or no [19] in 293 AD patients and 396 healthy controls [15], in 361 AD patients and 358 controls [16]. For the rs3785883 (within intron 3) SNP, it was found that there was significant association [13], or no [5, 14, 20]. For the rs1467967 (within exon 1) SNP, it was showed that there was significant association [5], or no [7, 11, 21]. There were consistent results on the association between the rs7521 [6, 7, 11, 22] and rs9468 [11] (too little data) SNPs and AD risk. Thus, these four SNPs of the *MAPT* gene were a matter of controversy.

Therefore, we conducted this meta-analysis to explore the association between the *MAPT* SNPs and AD risk. In summary, results from this meta-analysis suggest that of these SNPs tested, rs242557 is significantly associated with increased AD risk under the dominant genetic model, and the rs2471738 SNP is significantly associated with increased AD risk under all the four genetic model. In the stratified analysis by *APOE* ε4 allele status, *APOE* ε4 allele carriers, but not *APOE* ε4 allele non-carriers, were showed to be significantly associated with increased AD risk. This result indicates that there appears to be a gene-gene interaction between the *APOE* and the *MAPT* genes, which could increase susceptibility to AD. More studies should, however, be conducted to assess the interaction.

Because of the moderate heterogeneity, we conducted sensitivity analyses to evaluate the effects of each study on the combined ORs by sequential removal of each eligible study. The sensitivity analysis showed that none of these studies changed the significance of the combined ORs under the allelic model. It was showed that Allele A of rs242557 with the H1p promoter variant had 2.7-fold greater transcriptional activity than allele G with the H1p promoter variant and 4.2-fold greater than allele G with the H2p promoter variant. The H1 haplotype increases the expression of total *MAPT* transcript [6]; allele A (AA + AG) of rs242557 was associated with CSF total tau levels elevated levels compared to non-carriers (GG) [5], indicating that SNP rs242557 might be associated with the increased expression levels of tau protein. Trabzuni, D. et al [23]. found that the H1c haplotype (tagged by rs242557) was not significantly associated with increased mRNA expression of the *MAPT*, suggesting that there are other things about possible consequence of this SNP on the *MAPT*, which is needed for further investigations. In the current meta-analysis, SNP rs3785883 was found not to be associated with AD risk under all the genetic models; in AD cases, however, there was higher levels of Total tau mRNA in those individuals who carry rs3785883 minor allele (AA or AG) than those with non-carriers (GG) with evidence of beta-amyloid deposition [24], suggesting that SNP rs3785883, which changes the expression of the marker protein of AD, but is not associated with AD risk, might be an complicated SNP of the MAPT gene.

There are some limitations to this meta-analysis. First, the total number of studies was not large enough for such analyses to give meaningful interpretation, and only published studies were included in the meta-analysis. To be made, however, this approach requires the authors of all of the studies to share their data. Second, there was evidence of moderate heterogeneity between studies, in particularly for rs2471738. Third, the present meta-analysis failed to consider the possibility of gene-gene or SNP-SNP interactions in which further investigations are needed. So it is quite important to have more studies and sample in the future so that more precise conclusion about the association between the SNPs of the *MAPT* gene and AD risk could be achieved.

In conclusion, our meta-analysis confirmed the following: SNPs rs242557 and rs2471738 might be associated with increased AD risk, but rs3785883 and rs1467967 not. More well-conducted studies with larger sample size are needed to confirm our conclusion.

## MATERIALS AND METHODS

### Search strategies

All of the potential eligible studies were screened based on the electronic databases (PubMed and Google Scholar) up to 1st Jun. 2017. Systematic searching was performed using the combination of “Alzheimer*”, “rs242557 OR rs3785883 OR rs2471738 OR rs1467967 OR rs75721 OR rs9468”.

### Inclusion and exclusion criteria

Only studies published as full-length articles in peer-reviewed journals were considered in the analysis. The eligible studies must satisfy the following inclusion criteria: i) concerning the association between the *MAPT* gene (including SNPs rs242557, rs3785883, rs2471738, rs1467967, rs75721 and rs9468) and AD risk; ii) case-control study design; iii) sufficient information accessible (e.g. sample size for each study, allele or genotype frequencies of these SNPs); iv) cases meeting the clinical criteria for AD. The exclusion criteria include: a duplicated publication; a review; a case report; not reported the genotype frequencies; non-AD cases, a review; an irrelevant study; datum not available; an abstract; in neither English nor Chinese; inconsistent with most studies in major allele size.

### Data extraction

Data extracted from the included studies were as follows: first author, year of publication, country, sample size of cases and controls, numbers of case and control genotypes, *p-value* for HWE in controls and Newcastle-Ottawa Scale (NOS) Quality Assessment Scale. The inclusion/exclusion criteria were applied by 2 (ZFT and WDL) independent reviewers. We used the NOS to assess the quality of the included studies. A quality score was calculated based on three major components. Each component of the criteria scored 1 if present or 0 if absent. The scores were summed and a higher score represents better methodological quality.

### Meta-analysis

All statistical analyses were performed using Stata software (College Station, TX). The association between the *MAPT* SNPs and AD risk was evaluated by pooled ORs and corresponding 95% CIs. Four genetic models, including allelic (G *vs*. A), dominant (AA + AG *vs*. GG), recessive (AA *vs*. AG + GG) and additive (AA *vs*. GG), were used to estimate this association. Sensitivity analyses were performed to determine whether undue influence of a single study was present. The possibility of publication bias was assessed by Begg's and Egger's test (*P* < 0.05 was considered as representative of statistically significant publication bias).
